# Effect of statins on the association between high temperature and all-cause mortality in a socioeconomically disadvantaged population: a cohort study

**DOI:** 10.1038/s41598-019-41109-0

**Published:** 2019-03-18

**Authors:** Young Hee Nam, Warren B. Bilker, Charles E. Leonard, Michelle L. Bell, Lacy M. Alexander, Sean Hennessy

**Affiliations:** 1 Center for Pharmacoepidemiology Research and Training, Center for Clinical Epidemiology and Biostatistics, and Department of Biostatistics, Epidemiology and Informatics, Perelman School of Medicine, University of Pennsylvania, Philadelphia, PA 19104-4865 USA; 2 School of Forestry & Environmental Studies, Yale University, New Haven, CT 06511 USA; 3 Department of Kinesiology, College of Health and Human Development, The Pennsylvania State University, University Park, PA 16802 USA

**Keywords:** Health care, Epidemiology

## Abstract

High temperature increases all-cause mortality. Thermoregulatory ability is impaired in persons with elevated serum cholesterol, but can be improved by the administration of statins, even in the short-term. We investigated whether the impact of high temperature (≥24 °C) on all-cause mortality among socioeconomically disadvantaged adults with a current or past indication for a statin is attenuated by current use of a statin with temperature dependence, by using claims data from five US Medicaid programs supplemented with Medicare claims for dual-enrollees and meteorological data from 1999–2010. We identified 3,508,948 persons (3,181,752 person-years) in a 1:1 propensity score-matched cohort. The incidence rate of all-cause mortality (deaths per 1,000 person-years) was 21.9 (95% confidence interval [CI]: 21.6 to 22.3) in current statin users and 30.1 (95% CI: 30.2 to 30.6) in former users. The adjusted odds ratios of mortality for current vs. former statin use were statistically significantly lower than 1.0, suggesting a protective effect of current statin use, on days with high temperature, with either daily average temperature or daily maximum temperature, and declined as daily average temperature increased from 29 °C and daily maximum temperature increased from 34 °C. These results were robust to the adjustment for daily relative humidity.

## Introduction

There is a well-established U-shaped or similar relationship between ambient temperature and all-cause mortality^1,2^. Given this relationship, ongoing increases in global temperatures are expected to significantly increase mortality and morbidity, especially in vulnerable populations such as the socioeconomically disadvantaged, older adults, the frail, and those with chronic illnesses^2–5^. Humans’ ability to thermoregulate in response to high ambient temperatures depends largely on our ability to increase cutaneous blood flow in part through endothelial vasorelaxation^6,7^. Although this ability is impaired in persons with advanced age^8^ and those with elevated serum cholesterol^9^, it is improved by short-term administration of statins through mechanisms that appear to be independent of statins’ effects on lipids^10^. Statins improve cutaneous vascular function by stabilizing the essential cofactor tetrahydrobiopterin for the production of the vasoprotective molecule nitric oxide, which is needed for thermoregulatory cutaneous vasodilation^11^. Other potentially thermo-protective mechanisms of statins include vasoprotection^12^, endothelial function improvement^13,14^, and atherosclerotic plaque stabilization^13^. Given these effects, we hypothesized that statins might attenuate the association between elevated ambient temperature and all-cause mortality in socioeconomically disadvantaged populations, such as those enrolled in the United States Medicaid program, with temperature dependence.

## Methods

### Study design, population, and data

**Figure 1:**
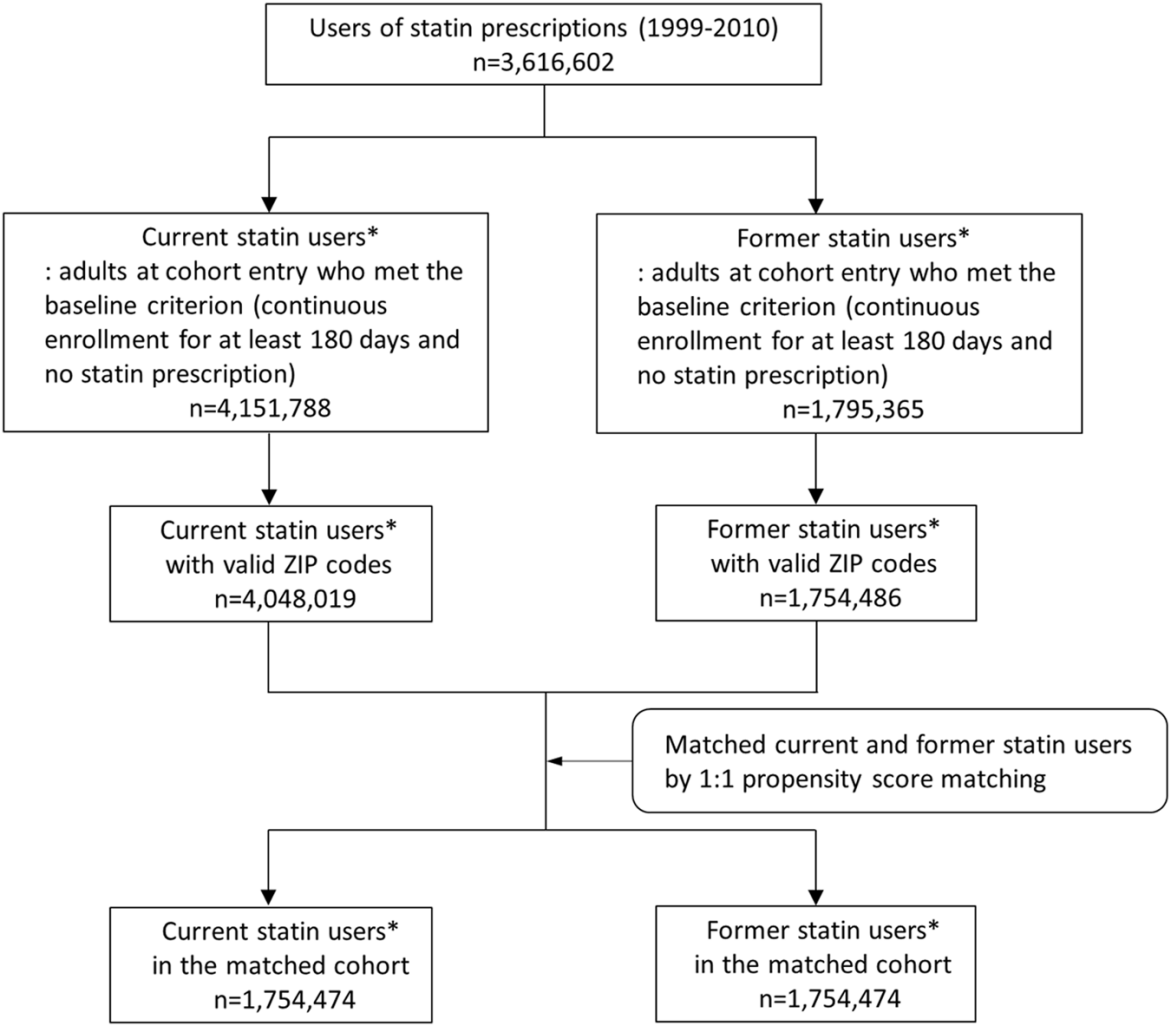
Construction of study cohort and sample size. *Number of individuals with distinct cohort entry date, not necessarily unique persons because individuals were included in the cohort as current users and/or former users multiple times if inclusion/exclusion criteria were met.

We conducted a propensity score-matched cohort study to compare the temperature dependence of all-cause mortality between statin initiators (while they continued to use statins) with that of former statin users who appeared to have stopped taking statins at least 180 days previously. The rationale for comparing statin initiators to former statin users was to reduce the potential healthy user bias encountered when comparing statin users to non-users^15–17^. The study population was the adult US Medicaid population residing in California, Florida, New York, Ohio, and Pennsylvania from 1999–2010. In the US, Medicaid, a public health insurance program, covers certain categories of low-asset persons including those with disabilities and older adults needing nursing home care. These five states account for about 40% of the US Medicaid population^18^. We supplemented Medicaid claims data with Medicare claims data for Medicaid-Medicare dual-enrollees. Adults between age of 18 and 100 years at cohort entry date (described below) who were enrolled in Medicaid continuously for at least 180 days immediately before the cohort entry (which constituted the baseline period) were eligible. The baseline period needed to be free of statin prescriptions so as to identify apparently new users^17^. Construction of the study cohort and sample sizes are shown in Fig. 1.

### Exposure and meteorological data

The parameter of interest was the multiplicative interaction between the exposure to statins and daily outdoor temperature, expressed separately as daily average temperature and daily maximum temperature. Study statins were all of the statins that were available in the US at any time during the study period: atorvastatin, cerivastatin, fluvastatin, lovastatin, pitavastatin, pravastatin, rosuvastatin, and simvastatin. Statins are available only by prescription in the US. The time of exposure to a statin was inferred for each prescription from the dispensing date and the days’ supply field.

Meteorological data were obtained from the US National Oceanic and Atmospheric Administration (NOAA)^19^, which reports weather parameters, including daily minimum and maximum temperatures measured at weather stations, and the locations of those stations. For each study individual, we linked the Zoning Improvement Plan (ZIP) code of residence (ascertained from the Medicaid data) to the population-weighted centroid of that ZIP code as estimated by using ZIP code boundaries, census block group boundaries, and 2010 census block group-level population data. Individuals with a missing or invalid ZIP code of residence were excluded. The daily average temperature (calculated as the arithmetic mean of the daily minimum and daily maximum temperatures) and daily maximum temperature for each population-weighted ZIP code centroid were estimated from daily meteorological data, locations of weather stations, and spline interpolation^20–22^.

### Study outcome and follow-up time

The outcome of interest was all-cause mortality, ascertained by the Social Security Administration Death Master File^23^.

For statin initiators, follow-up time (i.e., the current statin use period) began on the cohort entry date, defined as the dispensing date of the first statin prescription following the baseline period, and ended on the day of the following events, whichever occurred first: 1) death; 2) end of days’ supply of the statin prescription; 3) Medicaid enrollment discontinuation; or 4) end of the data set (i.e., December 31, 2010). We allowed a 7-day grace period at the end of each statin prescription (i.e., extended the days’ supply of each statin prescription by 7 days) to account for potential incomplete adherence, and considered consecutive statin prescriptions (which did not need to be for the same statin) without a gap longer than 7 days to be a continuous exposure to statins.

**Figure 2:**
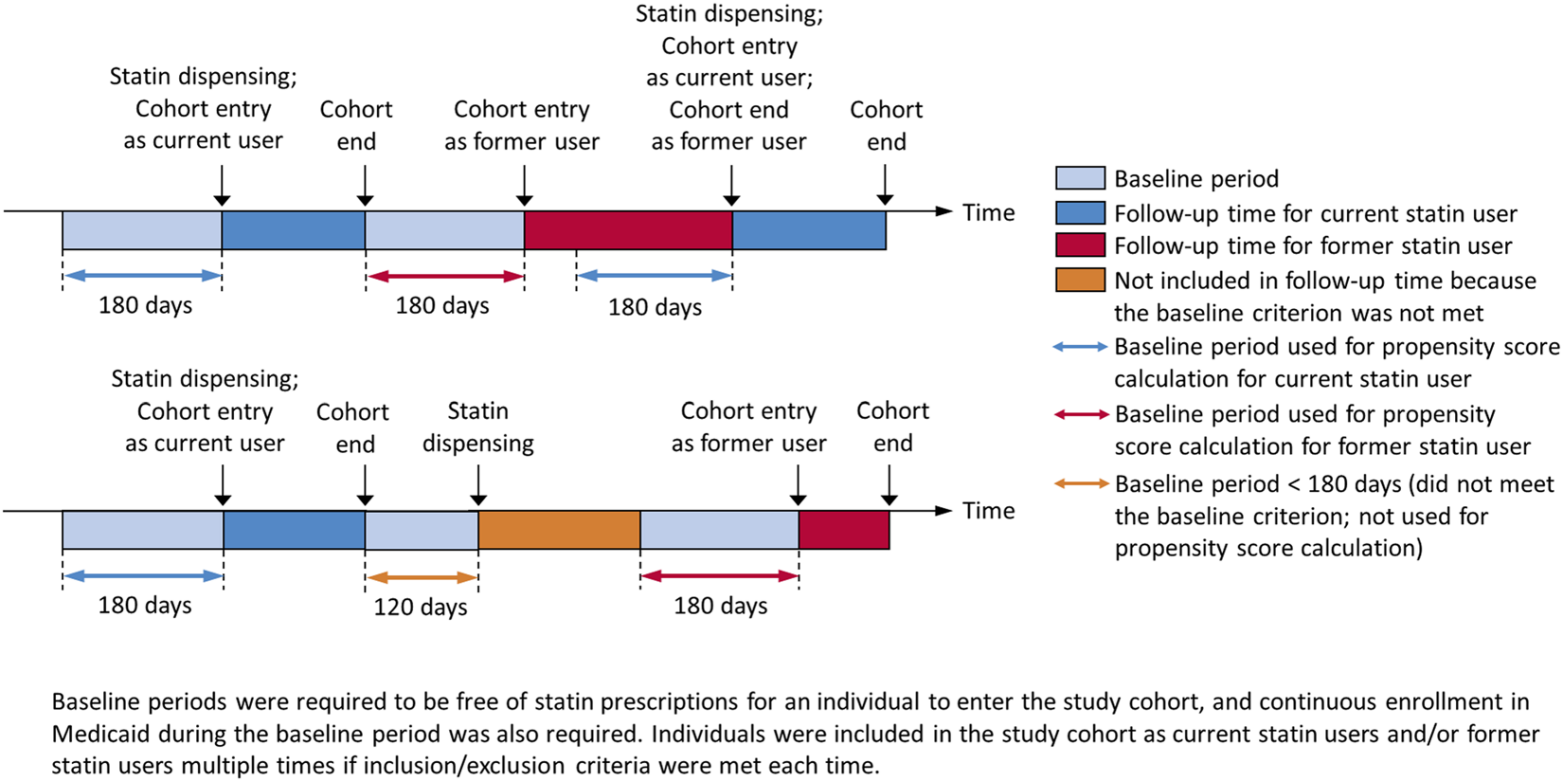
Follow-up time and baseline period.

To avoid conflating statins’ benefits with acute risks of statin discontinuation^24,25^, follow-up time for former statin users began 180 days after the end of the follow-up time of statin use. This 180-day period served as the baseline period for former statin users. Follow-up for this cohort ended on the day of the first occurrence of the following: 1) death; 2) dispensing of a statin prescription; 3) Medicaid enrollment discontinuation; or 4) end of the data set (i.e., December 31, 2010). Individuals were allowed to enter the cohort multiple times, as current and/or former statin users, if inclusion and exclusion criteria were met multiple times. Propensity scores were calculated for each cohort entry based on covariates assessed during the baseline period. Fig. 2 illustrates follow-up time and baseline period.

### Statistical analysis

#### Propensity score matching

We used propensity score matching to balance baseline covariates between statin initiators and former statin users^26,27^. We estimated each individual’s (i.e., each of the individuals with a distinct cohort entry date; not necessarily unique persons) propensity score for current statin use (i.e., calculated propensity score for each cohort entry) (Fig. 2) by fitting a logistic regression model where the dependent variable was the indicator of current vs. former statin use, and the independent variables were the baseline variables listed in Table 1, assessed during the baseline period. These baseline variables included: 1) demographic characteristics (including age, sex, race/ethnicity, state of residence, etc.); 2) diseases (including hypertensive disease, diabetes, etc.); 3) prescription drugs (including renin-angiotensin-aldosterone system blockers, beta-blockers, etc.); and 4) healthcare services utilization intensity (including nursing home residence, number of inpatient hospitalizations, number of outpatient visits, and number of prescription drug fillings)^28^. We then used 1:1 nearest neighbor propensity score matching (caliper of width = 0.01 on the propensity score scale) to match former statin users to statin initiators (who were more numerous). Observations within the same individual with a different exposure status were not preferentially matched to each other (i.e., all observations had the same probability to be matched based on propensity scores).

**Table 1:** Baseline characteristics of unmatched and propensity score-matched cohorts for all-cause mortality.

	Unmatched cohort			PS-matched cohort		
	Current statin users	Former statin users	Standardized Difference	Current statin users	Former statin users	Standardized Difference
	n = 4,048,019	n = 1,754,486		n = 1,754,474	n = 1,754,474	
***Sociodemographic Characteristics***						
Age at cohort entry, in years (%)						
18 ≤ Age <35	3.75	3.95	0.01	3.54	3.95	0.02
35 ≤ Age <50	19.05	18.94	0.00	18.08	18.94	0.02
50 ≤ Age <65	30.78	29.51	0.03	29.46	29.51	0.00
65 ≤ Age <80	35.32	35.74	0.01	37.12	35.74	0.03
80 ≤ Age <100	11.09	11.87	0.02	11.79	11.87	0.00
Sex, female (%)	62.73	63.97	0.03	64.81	63.97	0.02
Race/Ethnicity (%)						
White	39.34	35.83	0.07	35.15	35.83	0.01
Black	15.20	14.87	0.01	14.93	14.87	0.00
Hispanic	19.75	21.19	0.04	21.49	21.19	0.01
Other/Unknown	25.71	28.10	0.05	28.43	28.10	0.01
Medicaid-Medicare dual-eligible (%)	58.07	58.43	0.01	59.76	58.43	0.03
State of residence (%)						
CA	42.27	49.73	0.15	52.77	49.73	0.06
FL	14.14	8.79	0.17	7.11	8.79	0.06
NY	28.83	29.50	0.01	29.41	29.50	0.00
OH	8.27	7.07	0.05	6.38	7.07	0.03
PA	6.50	4.91	0.07	4.32	4.91	0.03
Urban residence^a^ (%)	89.80	91.12	0.04	91.35	91.12	0.01
Year of cohort entry (%)						
1999	2.01	0.00	0.20	0.00	0.00	0.00
2000	3.75	2.47	0.07	2.04	2.47	0.03
2001	4.73	4.44	0.01	4.39	4.44	0.00
2002	5.58	6.58	0.04	7.18	6.58	0.02
2003	6.48	7.47	0.04	8.12	7.47	0.02
2004	7.29	7.64	0.01	7.87	7.64	0.01
2005	8.69	9.52	0.03	9.81	9.52	0.01
2006	11.53	12.13	0.02	12.13	12.13	0.00
2007	9.69	13.45	0.12	15.13	13.44	0.05
2008	11.34	11.39	0.00	11.09	11.39	0.01
2009	13.28	11.71	0.05	10.73	11.71	0.03
2010	15.63	13.20	0.07	11.51	13.20	0.05
***Diseases***						
Anemia (%)	16.41	15.26	0.03	15.64	15.26	0.01
Artery and peripheral vascular diseases (%)	10.60	9.79	0.03	9.90	9.79	0.00
Arthritis (%)	16.52	16.99	0.01	17.99	16.99	0.03
Cancer (%)	6.32	6.31	0.00	6.57	6.31	0.01
Cardiac dysrhythmia/conduction disorders (%)	11.27	9.48	0.06	9.30	9.48	0.01
Cerebrovascular disease (%)	10.28	8.15	0.07	7.78	8.15	0.01
Chronic obstructive pulmonary disease (%)	16.05	15.40	0.02	15.92	15.40	0.01
Coagulation defect (%)	1.06	0.98	0.01	0.99	0.98	0.00
Depression (%)	6.30	6.50	0.01	6.86	6.50	0.01
Diabetes (%)	34.08	30.44	0.08	30.45	30.44	0.00
Fluid/electrolyte disorders (%)	7.13	6.73	0.02	6.84	6.73	0.00
Heart failure (%)	9.73	8.37	0.05	8.26	8.37	0.00
HIV/AIDS (%)	1.10	1.04	0.01	1.09	1.04	0.00
Hypertensive diseases (%)	52.33	46.39	0.12	46.11	46.39	0.01
Hypothyroidism (%)	9.94	8.33	0.06	8.23	8.33	0.00
Ischemic heart diseases (%)	20.96	16.79	0.11	16.13	16.79	0.02
Liver disease (%)	3.37	3.71	0.02	4.02	3.71	0.02
Neurological disorders (%)	11.31	10.57	0.02	10.84	10.57	0.01
Peptic ulcer diseases (%)	1.40	1.55	0.01	1.68	1.55	0.01
Psychosis (%)	5.38	5.37	0.00	5.65	5.37	0.01
Pulmonary circulation disease (%)	1.58	1.22	0.03	1.15	1.22	0.01
Renal failure (%)	6.14	5.81	0.01	5.89	5.81	0.00
Valvular heart diseases (%)	7.08	5.26	0.08	4.89	5.26	0.02
***Prescription Drugs***						
Adrenergic drugs (%)	7.76	7.49	0.01	7.75	7.49	0.01
Antiarrhythmic drugs (%)	1.01	0.95	0.01	0.97	0.95	0.00
Anticancer drugs (%)	2.36	2.34	0.00	2.45	2.34	0.01
Anticoagulants (%)	4.33	3.71	0.03	3.70	3.71	0.00
Anticonvulsants (%)	5.89	5.57	0.01	5.77	5.57	0.01
Antidepressants (%)	26.75	24.77	0.05	25.27	24.77	0.01
Antidiabetes drugs (%)	30.11	27.52	0.06	27.68	27.52	0.00
Anti-infectives (%)	40.91	41.59	0.01	43.54	41.59	0.04
Antiplatelets (%)	1.28	1.13	0.01	1.12	1.13	0.00
Antipsychotics (%)	11.24	11.24	0.00	11.84	11.24	0.02
Antiretrovirals (%)	0.99	0.95	0.00	1.00	0.95	0.01
Beta-blockers (%)	24.28	21.74	0.06	21.78	21.74	0.00
Bronchodilators/inhaled corticosteroids (%)	15.97	15.65	0.01	16.27	15.65	0.02
Calcium channel blockers (%)	23.01	21.55	0.04	22.04	21.55	0.01
Corticosteroids (%)	14.07	14.06	0.00	14.70	14.06	0.02
Diuretics (%)	29.61	26.33	0.07	26.33	26.33	0.00
Inotropic (%)	3.11	2.71	0.02	2.74	2.71	0.00
Leukotriene (%)	3.91	4.07	0.01	4.30	4.07	0.01
MAOIs	0.05	0.05	0.00	0.05	0.05	0.00
NSAIDs	34.09	34.01	0.00	35.34	34.01	0.03
Potassium supplements (%)	6.80	5.95	0.03	5.96	5.95	0.00
PPIs, H2 antagonists	32.03	31.76	0.01	33.08	31.76	0.03
RAAS blockers	34.05	30.07	0.09	29.82	30.07	0.01
Thyroid hormones	9.14	8.41	0.03	8.57	8.41	0.01
Vasodilators	9.09	7.95	0.04	7.98	7.95	0.00
Xanthine derivatives (%)	0.91	0.78	0.01	0.79	0.78	0.00
***Healthcare Services Utilization Intensity***						
Nursing home residence (%)	6.62	5.60	0.04	5.49	5.60	0.00
Inpatient hospitalization, mean number	0.27	0.22	0.04	0.20	0.22	0.02
Outpatient visits, mean number	19.86	20.52	0.01	20.65	20.52	0.00
Prescription drug fillings, mean number	10.39	10.03	0.02	10.38	10.03	0.02

PS-matched cohort: propensity score-matched cohort. MAOIs: monoamine oxidase inhibitors. PPIs: proton pump inhibitors. RAAS: renin-angiotensin-aldosterone system. Ref: reference. ^a^Urban residence: ascertained by the ZIP codes in the claims data used and ZIP Code to Carrier Locality File from the Centers for Medicare and Medicaid Services (Centers for Medicare and Medicaid Services, 2017). A participant can have more than one diagnosis and/or more than one prescription.

#### Baseline characteristics, incidence rates of outcome, and measures of association

We first tabulated descriptive statistics on baseline characteristics and compared mortality rates between statin initiators and former statin users, both before and after propensity-score matching. Balance in baseline characteristics was assessed by standardized difference (i.e., the mean difference of a variable between the two groups in units of the estimated common standard deviation), with a value greater than 0.1 suggestive of potentially meaningful imbalance between groups^27^.

We then used logistic regression to examine the effect of interaction between temperature on days in the high temperature range (defined as ≥24 °C) and current vs. former statin use on all-cause mortality. The unit of observation was person-day, and each temperature metric was assessed each day for each individual. We assessed the temperature dependence of current statin use, the main parameter of our interest, by modeling the interaction between temperature (daily average temperature and daily maximum temperature were used in the separate models) and statin exposure. The threshold minimum temperature of 24 °C was chosen based on literature indicating a general U-shape or similar relationship between temperature and mortality, with a nadir (often called the minimum mortality temperature) between 22–26 °C, although we recognized that this relationship may vary by location and/or health outcome^29–32^. We excluded observations where daily average temperature exceeding 43 °C and where daily maximum temperature exceeding 49 °C because there were few observations above these thresholds to yield stable models. Since the true functional forms of the relationship between temperature, statin use, and the outcome are unknown, we fitted a logistic regression model that included both a linear term and a quadratic term for temperature, and two temperature-by-current-statin-use interaction terms, in addition to the current statin use indicator. Inclusion of a quadratic term avoided reliance on the assumption that the relationship between temperature and outcome occurrence was linear. This model is expressed as Equation 1.

1 $$\text{logit}({Y}_{ij})=\alpha +{\beta }_{0}({T}_{ij})+{\beta }_{1}({T}_{ij}^{2})+{\beta }_{2}({C}_{i})+{\beta }_{3}({T}_{ij}\times {C}_{i})+{\beta }_{4}({T}_{ij}^{2}\times {C}_{i})+\,{{\epsilon }}_{ij}$$

In this equation, *Y*_*ij*_ is an indicator variable for the outcome occurrence of person *i* on day *j*; *T*_*ij*_ is the outdoor temperature (either daily average temperature or daily maximum temperature) for person *i* at their ZIP code area on day *j*; and *C*_*i*_ is a binary variable indicating current vs. former use of statin of person *i*. In a sensitivity analysis, we examined a model that additionally included relative humidity on each day at the person level.

Analyses were performed by using ArcGIS version 10.3 (Esri, Redlands, California, USA), SAS version 9.4 (SAS Institute Inc., Cary, North Carolina, USA), and Stata version 14 (StataCorp, College Station, Texas, USA).

### Ethical Approval

This study was approved by the institutional review board of the University of Pennsylvania, which waived the requirement for obtaining informed consent. All methods were carried out in accordance with the relevant guidelines and regulations. We attest that we have obtained appropriate permissions and paid any required fees for use of any copyright protected materials.

## Results

Prior to matching, we identified 4,048,019 current statin users and 1,754,486 former statin users who met inclusion and exclusion criteria. After 1:1 matching, each of the current and former statin user groups included 1,754,474 individuals (Table 1). Table 1 presents baseline characteristics of the current and former statin user groups before and after matching. Before matching, the groups were reasonably well balanced on baseline characteristics, although hypertensive diseases and ischemic heart diseases were more common in statin initiators than in former users. Balance was improved by matching, as suggested by all post-matching standardized differences being less than 0.1.

Median follow-up times of current and former statin users after matching were 69 and 290 days, respectively. The mortality rate in deaths per 1,000 person-years in the matched cohort was 21.9 (95% confidence interval [CI]: 21.6 to 22.3) in current statin users and 30.4 (95% CI: 30.2 to 30.6) in former statin users (Table 2), showing an overall rate ratio for current vs. former statin use of 0.72 (95% CI: 0.72 to 0.73).

**Table 2:** Incidence rates of all-cause mortality in unmatched and propensity score-matched cohorts.

	Number of persons	Number of deaths	Person-years	Mortality rate in deaths per 1,000 person-years (95% Confidence Interval)
Unmatched cohort				
Overall	5,802,505	113,545	4,052,108	28.0 (27.9 to 28.2)
Current statin users	4,048,019	37,416	1,549,524	24.1 (23.9 to 24.4)
Former statin users	1,754,486	76,129	2,502,584	30.4 (30.2 to 30.6)
PS-matched cohort				
Overall	3,508,948	91,020	3,181,752	28.6 (28.4 to 28.8)
Current statin users	1,754,474	14,892	679,179	21.9 (21.6 to 22.3)
Former statin users	1,754,474	76,128	2,502,573	30.4 (30.2 to 30.6)

PS-matched cohort: propensity score-matched cohort.

**Figure 3:**
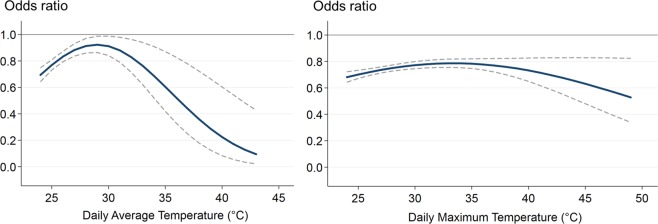
Odds ratios and 95% confidence intervals of all-cause mortality for current vs. former use of statins by temperature. Daily average temperature: 24–43 °C (75–110 °F). Daily maximum temperature: 24–49 °C (75–120 °F). Bold solid lines indicate odds ratios, and thin dash lines indicate 95% confidence intervals.

**Figure 4:**
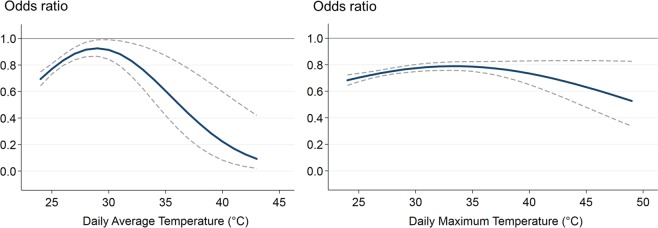
Odds ratios and 95% confidence intervals of all-cause mortality for current vs. former use of statins by temperature, additionally controlling for daily relative humidity. Daily average temperature: 24–43 °C (75–110 °F). Daily maximum temperature: 24–49 °C (75–120 °F). Bold solid lines indicate odds ratios, and thin dash lines indicate 95% confidence intervals.

Table 3 presents the results of the logistic regression, and Fig. 3 plots the odds ratios (with 95% CIs) of all-cause mortality for current vs. former statin use by temperature (calculated based on the regression results). As shown in Fig. 3, throughout the high temperature range included in the analysis (≥24 °C), the odds ratios were statistically significantly less than 1.0 for both daily average temperature and daily maximum temperature, suggesting a protective effect of current statin use. The interaction terms of current statin use and temperature, the main parameters of interest, were statistically significant: *p* = 0.0001 with daily average temperature, *p* = 0.0003 with daily average temperature squared; *p* = 0.009 with daily maximum temperature, and *p* = 0.016 with daily maximum temperature squared (Table 3). With both daily average temperature and daily maximum temperature, the odds ratios declined (i.e., the protective effect of current statin use increased) as daily average temperature increased from 29 °C and daily maximum temperature increased from 34 °C (Fig. 3). This estimated relationship corresponds to approximately a 6% point reduction in the odds ratio of mortality for each 1 °C increase in daily average temperature between 29 °C and 43 °C, and a 2% point reduction in the odds ratio for mortality for each 1 °C increase in daily maximum temperature between 34 °C and 49 °C. Results were similar when daily relative humidity was included in the regression model (Table 4 and Fig. 4).

**Table 3:** Results of logistic regression assessing the association of all-cause mortality with temperature, statins, and their interaction.

	Daily average temperature				Daily maximum temperature			
	(n = 195,222,132 person-days; 15,771 deaths)				(n = 503,398,992 person-days; 40,280 deaths)			
	Coefficient^a^	95% CI		*p-*value	Coefficient	95% CI		*p-*value
		Lower	Upper			Lower	Upper	
Intercept	−9.8674	−11.4985	−8.2363	<0.0001	−8.9603	−9.4481	−8.4725	<0.0001
Temperature	0.0198	−0.0984	0.1380	0.743	−0.0342	−0.0661	−0.0023	0.036
Temperature squared^b^	−0.0001	−0.0022	0.0021	0.960	0.0007	0.0002	0.0012	0.011
Statin^c^	−9.8240	−14.5415	−5.1065	<0.0001	−2.0564	−3.3039	−0.8089	0.001
Temperature × Statin	0.6729	0.3283	1.0175	0.0001	0.1088	0.0269	0.1907	0.009
Temperature squared × Statin	−0.0116	−0.0179	−0.0053	0.0003	−0.0016	−0.0030	−0.0003	0.016

95% CI: 95% confidence interval. Daily average temperature: 24–43 °C (75–110 °F). Daily maximum temperature: 24–49 °C (75–120 °F). *p*-value: α = 0.05, two-tailed test. ^a^Coefficient: coefficients in the logistic regression analysis. ^b^Temperature squared: 2nd degree polynomial term of temperature. ^c^Statin: current statin exposure status (1 = current statin user; 0 = former statin user).

**Table 4:** Sensitivity analysis: Statins’ effect on the association between high temperature and all-cause mortality: results of logistic regression with daily relative humidity included.

	Daily average temperature			Daily maximum temperature				
	(n = 195,222,132 person-days; 15,771 deaths)			(n = 503,398,992 person-days; 40,280 deaths)				
	Coefficient^a^	95% CI		*p-*value	Coefficient	95% CI		*p-*value
		Lower	Upper			Lower	Upper	
Intercept	−9.9519	−11.5942	−8.3096	<0.0001	−8.9466	−9.4384	−8.4548	<0.0001
Temperature	0.0341	−0.0851	0.1533	0.575	−0.0141	−0.0464	0.0181	0.395
Temperature squared^b^	−0.0004	−0.0026	0.0018	0.750	0.0002	−0.0003	0.0007	0.359
Statin^c^	−9.9122	−14.6399	−5.1845	<0.0001	−2.0752	−3.3320	−0.8184	0.001
Temperature × Statin	0.6791	0.3337	1.0245	0.0001	0.1102	0.0277	0.1927	0.009
Temperature squared × Statin	−0.0117	−0.0180	−0.0054	0.0003	−0.0017	−0.0030	−0.0004	0.016
Relative humidity^d^	−0.0016	−0.0026	−0.0006	0.002	−0.0042	−0.0049	−0.0035	<0.0001

95% CI: 95% confidence interval. Daily average temperature: 24–43 °C (75–110 °F). Daily maximum temperature: 24–49 °C (75–120 °F). *p*-value: α = 0.05, two-tailed test. ^a^Coefficient: coefficients in the logistic regression analysis. ^b^Temperature squared: 2nd degree polynomial term of temperature. ^c^Statin: current statin exposure status (1 = current statin user; 0 = former statin user). ^d^Relative humidity: daily relative humidity.

## Discussion

Climate change is raising overall temperature and the frequency and magnitude of temperature extremes, and thereby increasing heat-related mortality and morbidity, especially among the socioeconomically disadvantaged and other vulnerable populations^2–5^. Humans’ vasodilatory response to elevated skin temperature is improved by even short-term administration of statins^33,34^. We therefore hypothesized that statins might attenuate the well-established relationship between high temperature and all-cause death, and this association of statins might be temperature dependent. We found that current statin use had apparent protective effect against all-cause mortality in a socioeconomically disadvantaged population of ever-users of statins, and that this survival benefit of statins strengthened (i.e., the odds ratio of mortality for current vs. former statin use became smaller) as daily average temperature and daily maximum temperature increased. These relationships appear to be robust to inclusion of daily relative humidity. Our findings provide initial epidemiologic support for the physiologically-based hypothesis that statins might confer protection against heat-related death.

### Strengths and limitations of the current study

This study has several strengths, including the use of large data, linkage to daily meteorological data, inclusion of groups that were well balanced in baseline characteristics, and use of an unambiguous endpoint. This study also has limitations. First, we do not know the degree to which individuals were actually exposed to outdoor temperatures, because data on individuals’ use of air conditioning or the amount of time spent outdoors were unavailable. However, given that all participants were enrolled in Medicaid, a public health insurance program for those who meet certain low-socioeconomic status criteria, it seems unlikely that access to air conditioning was markedly imbalanced between current and former statin users. Second, despite the observed balance on measured factors, possibility of the difference in unmeasured susceptibility to heat cannot be excluded. To reduce this risk of comparing the temperature dependence of mortality across possibly inherently incomparable groups (as might occur were we to compare statin users to non-users, because of the healthy statin user effect^35^), we compared statin initiators to former users who were matched on a propensity score. However, exact reasons why individuals ceased the use of statins are unknown from our data, which is a potential confounder. Nearly half of statin initiators are known to discontinue use within a year^36–38^, and older age, vascular comorbidity, and obesity are inversely associated with discontinuation^39^, which would reduce the protective association of statins. Indeed, the few pre-matching imbalances that we observed would have disadvantaged the current statin user group. Third, the association between temperature and mortality, as well as statins’ effect on the association between temperature and mortality, can differ by geographic location and/or demographic subgroup. These relationships will need to be investigated in future work. Finally, these results observed in current and former users of statins in the US Medicaid population might not be generalizable to those without a clinical indication for statins, or to socioeconomically less vulnerable populations^40^.

### Comparison with other studies

The overall association between current statin use and all-cause mortality (i.e., the ratio of adjusted all-cause mortality rates of current vs. former statin use) in this study was 0.72 (95% CI: 0.72 to 0.73), which is closer to that found in a network meta-analysis of randomized statin trials (odds ratio, 0.87; 95% CI: 0.82 to 0.92)^41^ than to that found in a recent observational cohort study comparing statin use to non-use (hazard ratio: 0.45; 95% CI: 0.40 to 0.50)^40^. We are aware of no prior studies that have examined a statin effect on the temperature dependence of death.

### Clinical and policy implications

Climate change is resulting in a greater exposure to potentially harmful temperatures with higher overall temperatures and more frequent extremes, and vulnerable populations are particularly susceptible to health effects of heat. Based on statins’ demonstrated effects in improving humans’ cutaneous vasodilatory response to heat, they might be expected to provide a thermo-protection to high-risk persons. This study suggests that in socioeconomically disadvantaged persons with a clinical indication for statins, the use of statins, even acutely, may confer survival benefit on hot days. This initial epidemiologic evidence deserves to be confirmed in further studies, and additional questions remain. Is the benefit restricted to those with a traditional clinical indication for statins? Do similar benefits accrue to those who are not socioeconomically disadvantaged? Does the apparent benefit vary by individual statin, stain dose, geography, duration of heat waves, availability of air conditioning, or other factors? Are there adverse effects of statin use on other heat-related health outcomes? While it may be feasible to implement systems that remind patients to take their statin on hot days, it may be premature to implement such systems before these questions are addressed. The most effective way to reduce harmful heat effect on mortality might be to avoid exposure to heat, if it is possible. When this strategy is not possible, statins’ thermo-protective effect might be beneficial to some of those with an indication for a statin and exposed to high temperatures.

## Conclusion

This study found that in a socioeconomically disadvantaged population, current statin users had lower odds of all-cause mortality than former statin users in the high temperature range, and this protective association of statins increased as daily average temperature and daily maximum temperature increased. Individuals with a statin indication might benefit even more from taking their statin on hot days. Further studies are warranted to confirm and clarify this relationship.

## Data Availability

The US Medicaid and Medicare claims are available to obtain under a data use agreement from the Centers for Medicare & Medicaid Services (CMS) (https://www.cms.gov/). The procedures to obtain access to these data are described in the CMS website (https://www.cms.gov/Research-Statistics-Data-and-Systems/Research/ResearchGenInfo/ResearchDataAssistanceCenter.html) and the Research Data Assistance Center (ResDAC) website (https://www.resdac.org/cms-data/request/cms-data-request-center).
