# Publisher Correction: Effect of statins on the association between high temperature and all-cause mortality in a socioeconomically disadvantaged population: a cohort study

**DOI:** 10.1038/s41598-019-46259-9

**Published:** 2019-07-24

**Authors:** Young Hee Nam, Warren B. Bilker, Charles E. Leonard, Michelle L. Bell, Lacy M. Alexander, Sean Hennessy

**Affiliations:** 1 Center for Pharmacoepidemiology Research and Training, Center for Clinical Epidemiology and Biostatistics, and Department of Biostatistics, Epidemiology and Informatics, Perelman School of Medicine, University of Pennsylvania, Philadelphia, PA 19104-4865 USA; 2 School of Forestry & Environmental Studies, Yale University, New Haven, CT 06511 USA; 3 Department of Kinesiology, College of Health and Human Development, The Pennsylvania State University, University Park, PA 16802 USA

Correction to: *Scientific Reports* 10.1038/s41598-019-41109-0, published online 18 March 2019

This Article contains an error in Table 4. The *p*-values recorded for the ‘Daily average temperature column’ are incorrectly placed under the ‘Daily maximum temperature’ column. The correct Table 4 appears below as Table 1.

**Table 1:** Sensitivity analysis: Statins’ effect on the association between high temperature and all-cause mortality: results of logistic regression with daily relative humidity included.

	Daily average temperature				Daily maximum temperature			
	(n = 195,222,132 person-days; 15,771 deaths)				(n = 503,398,992 person-days; 40,280 deaths)			
	Coefficient^a^	95% CI		*p-*value	Coefficient	95% CI		*p-*value
		Lower	Upper			Lower	Upper	
Intercept	−9.9519	−11.5942	−8.3096	<0.0001	−8.9466	−9.4384	−8.4548	<0.0001
Temperature	0.0341	−0.0851	0.1533	0.575	−0.0141	−0.0464	0.0181	0.395
Temperature squared^b^	−0.0004	−0.0026	0.0018	0.750	0.0002	−0.0003	0.0007	0.359
Statin^c^	−9.9122	−14.6399	−5.1845	<0.0001	−2.0752	−3.3320	−0.8184	0.001
Temperature $\times$ Statin	0.6791	0.3337	1.0245	0.0001	0.1102	0.0277	0.1927	0.009
Temperature squared $\times$ Statin	−0.0117	−0.0180	−0.0054	0.0003	−0.0017	−0.0030	−0.0004	0.016
Relative humidity^d^	−0.0016	−0.0026	−0.0006	0.002	−0.0042	−0.0049	−0.0035	<0.0001

95% CI: 95% confidence interval. Daily average temperature: 24–43 °C (75–110 °F). Daily maximum temperature: 24–49 °C (75–120 °F). *p*-value: α = 0.05, two-tailed test. ^a^Coefficient: coefficients in the logistic regression analysis. ^b^Temperature squared: 2nd degree polynomial term of temperature. ^c^Statin: current statin exposure status (1 = current statin user; 0 = former statin user). ^d^Relative humidity: daily relative humidity.

